# Transcriptome analysis reveals regulatory mechanism of methyl jasmonate-induced monoterpenoid biosynthesis in *Mentha arvensis* L.

**DOI:** 10.3389/fpls.2024.1517851

**Published:** 2025-01-15

**Authors:** Tingting Huang, Wenjin Men, Ariuntungalag Myanganbayar, Undarmaa Davaasambuu

**Affiliations:** Laboratory of Applied Biological Control, School of Agroecology, Mongolian University of Life Sciences, Ulaanbaatar, Mongolia

**Keywords:** *Mentha arvensis* (mint), essential oil, jasmonic acid, monoterpenoid, glandular trichome, transcriptome

## Abstract

*Mentha arvensis* L. (*M. arvensis*) is an aromatic plant of the *Mentha* genus, renowned for its medicinal and economic importance. The primary components of its essential oils (EOs) are monoterpenoids, synthesized and stored in peltate glandular trichomes (PGTs). In general, the EO content in *M. arvensis* is relatively low. Methyl jasmonate (MJ) has been reported as an effective elicitor of terpenoid biosynthesis in medicinal plants, but the specific mechanisms underlying MJ’s influence on *M. arvensis* remain unclear. In this study, exogenous application of MJ significantly increased the EO content, yield, and PGT density in a dose-dependent manner. At a 5 mM dose, the EO content and PGT density peaked, with increases of 71.20% and 53.69%, respectively. Gas chromatography-mass spectrometry (GC-MS) analysis indicated that, in general, MJ treatment did not significantly alter the types or relative proportions of EO components of *M. arvensis*. However, L-menthol content decreased slightly by 7.90% under 5 mM MJ treatment. Transcriptome analysis identified 4,659 differentially expressed genes (DEGs) in MJ-treated leaves. KEGG enrichment analysis revealed that “Monoterpenoid biosynthesis” was among the most significantly enriched metabolic pathways. Key genes involved in jasmonic acid (JA) signaling (*JAZs* and *MYCs*) and monoterpenoid biosynthesis (*GPPSs*, *LSs*, *L3Hs*, and *IPRs*) were significantly up-regulated. Co-expression analysis, promoter binding element analysis and weighted gene co-expression network analysis (WGCNA) indicated that transcription factors (TFs) such as AP2/ERF, WRKY, MYB, and bHLH play crucial roles in regulating MJ-mediated monoterpenoid biosynthesis. Several key candidate TFs potentially involved in regulating monoterpenoid biosynthesis in *M. arvensis* were identified. These findings provide valuable insights into the molecular mechanisms regulating monoterpenoid accumulation in the *Mentha* genus.

## 1 Introduction

*Mentha arvensis* L., commonly known as Japanese mint, is widely distributed across various regions of the world. Its geographical range mainly includes Asia, Europe, and North America (Kumar et al., 2012; Vining et al., 2020). Cultivated primarily for the production of essential oil (EO), this plant thrives in moist, temperate climates and is often found in wetlands, riverbanks, and other water-abundant areas (Bussmann et al., 2020). The EO of *Mentha* species is widely utilized in medicine, cosmetics, food and other industries. It is primarily composed of monoterpenoids such as L-menthol, neomenthol, isomenthol, and carvone, which are synthesized and stored in peltate glandular trichomes (PGTs) of above-ground plant tissues (Ahkami et al., 2015; Lange, 2015). In *M. arvensis*, L-menthol is the predominant monoterpenoid, accounting for more than 70% of the total EO content (Batool et al., 2020). This compound is highly sought after in global markets for its cooling and fragrance properties, making it a key natural product in pharmaceutical and cosmetic applications (Khaliq and Mushtaq, 2023). However, the EO content in *Mentha* plants is relatively low, and the production does not meet the growing demand in pharmaceutical and other industries (Riaz et al., 2021).

The biosynthesis of terpenoids involves a series of biochemical processes, including precursor formation, intermediate conversion, end product generation, and post-modification (Mahmoud and Croteau, 2002). In mint, monoterpenoid biosynthesis starts with the methylerythritol phosphate (MEP) pathway, where pyruvate and 3-phosphoglyceraldehyde are converted into isopentenyl diphosphate (IPP) via six enzymatic steps. Key enzymes in this process include 1-deoxy-D-xylulose-5-phosphate synthase (DXS) and 1-deoxy-D-xylulose-5-phosphate reductoisomerase (DXR). IPP is then partially converted to dimethylallyl diphosphate (DMAPP) by isopentenyl diphosphate isomerase (IPPI), and these isomers combine through geranyl diphosphate synthase (GPPS) to form geranyl diphosphate (GPP) (Ahkami et al., 2015; Mahmoud and Croteau, 2002). Subsequently, GPP is converted to limonene by limonene synthase (LS), then catalyzed by limonene-3-hydroxylase (L3H), isopiperitenol dehydrogenase (IPD), isopiperitenone reductase (IPR), pulegone reductase (PR), and L-menthol reductase (MD), etc., resulting in the formation of valuable secondary metabolites such as menthone, isomenthone, and L-menthol (Mahmoud and Croteau, 2002; Croteau et al., 2005). Earlier studies on the *Mentha* genus primarily focused on identifying active compounds and conducting bioactivity assays (Zeljkovi et al., 2020; Mamadalieva et al., 2020). Recent research highlights the roles of structural genes in terpenoid biosynthesis. For instance, in *Mentha piperita*, overexpression of *DXS* and *DXR* and *IPPI* enhances its EO content by about 15%, 60% and 26%, respectively (Lange et al., 2011; Mahmoud and Croteau, 2001, 2002; Lange et al., 2011). While overexpression of *GPPS* enhanced *Mentha piperita* EO by 18% and increased tobacco limonene content approximately 35-fold (Yin et al., 2017; Lange et al., 2011).

Strategies to enhance terpenoid production now focus on modulating biosynthesis pathways and increasing the density or size of PGTs (Pandey et al., 2021; Pribat et al., 2013; Singh et al., 2024). Jasmonic acid (JA), a key phytohormone, plays a crucial role in plant defense and stress responses (Wang et al., 2020), while also regulating secondary metabolite synthesis and secretory structure development by reprogramming gene expression (Maes et al., 2011; Wasternack and Hause, 2013). For example, JA and its derivatives methyl jasmonates (MJ) promote terpenoid synthesis and PGT development in medicinal plants like *Artemisia annua* (*A. annua*), *Isodon rubescens*, and *Chrysanthemum indicum* var. *aromaticum* (Maes et al., 2011; Li, 2015; Zhang, 2021). High-throughput RNA sequencing (RNA-seq) has been extensively utilized to elucidate the molecular mechanisms of MJ in regulating terpenoid biosynthesis in plants. In *Pogostemon Cablin*, MJ treatment significantly increased the content of sesquiterpene patchouli alcohol, through upregulation of MEP pathway genes (Chen et al., 2019). In *Sindora glabra*, MJ increased the content of the sesquiterpenes α-copaene and β-caryophyllene, leading to the upregulation of most mevalonate (MVA) pathway genes, while the majority of MEP pathway genes were significantly down-regulated (Yu et al., 2021a). Although MJ treatment led to an increase in terpenoid content both in *Sindora glabra* and *Pogostemon Cablin*, the response patterns of structural genes in the MVA and MEP pathways differed between the two species. These findings highlight the species-specific mechanisms in JA-regulated terpenoid biosynthesis.

Transcription factors (TFs) induced by MJ are pivotal in regulating terpenoid biosynthesis and PGT development, such as AP2/ERF (apetala2/ethylene-responsive factor), bHLH (basic/helix-loop-helix), MYB (myeloblastosis DNA-binding protein), WRKY (WRKY-type DNA binding protein), and NAC (NAM, ATAF, and CUC domain protein) family genes. For example, the AP2/ERF family TF CrORCA3 in *Catharanthus roseus* and AaERF1 in *A. annua* positively regulate terpenoid synthesis by activating structural gene promoters (van der Fits and Memelink, 2000; Tan et al., 2015; Yu et al., 2012). Similarly, the bHLH TF MYC2 is a central regulator of JA signaling, coordinating transcriptional networks across multiple pathways. CrMYC2, induced by JA, promotes the biosynthesis of vinblastine by activating the transcription of *CrORCA3*, thereby actively regulating vinblastine synthesis (Pan et al., 2012; Sui et al., 2018). In *A. annua*, AaMYC2 can directly activate the expression of artemisinin synthesis structural genes, or actively regulate artemisinin synthesis through the MYC2-GSW1 (WRKY)-ORA (AP2/ERF) transcription cascade regulatory module (Chen et al., 2017; Shen et al., 2016). Negative regulators like JAZ proteins repress terpenoid biosynthesis by inhibiting TFs or structural genes. In *A. annua*, 9 AaJAZs bind to *AabHLH1* and inhibit its activation of the artemisinin biosynthesis structural genes *AaADS* and *AaCYP71AV1* (Li et al., 2019). Overexpression of *AaJAZ8* significantly reduces the PGT density, AaJAZ8 negatively regulates the initiation of PGT by inhibiting the expression of *AaSEP1* and *AaHD1*, thereby reducing artemisinin accumulation. When JAs and light are sufficient, AaJAZ8 is degraded by the 26S proteasome system, releasing AaSEP1, and promoting the activation of the AaSEP1-AaMYB16-AaHD1-AaGSW2 transcriptional cascade regulatory module (Chen et al., 2023; Xie et al., 2021a). Despite these advances, the regulatory mechanisms underlying monoterpenoid biosynthesis and PGT formation remain poorly understood.

In this study, MJ was used as an exogenous hormone to investigate its effects on the EO content, yield, and the PGT density in *M*. a*rvensis*. Gas chromatography-mass spectrometry (GC-MS) was used to analyze chemical components in the EO. High-throughput RNA sequencing was performed to identify potential molecular regulatory mechanisms of MJ in regulating monoterpenoid biosynthesis of mint. This research provides new insights into molecular basis of monoterpenoid biosynthesis and lays the groundwork for further studies in *Mentha* species.

## 2 Materials and methods

### 2.1 Plant cultivation and long-term plant hormone treatment for essential oil and peltate glandular trichome analysis

A commercial variety of *M. arvensis* was propagated by planting root segments under natural conditions. Each root segment contained one growth point and was planted in a substrate composed of peat soil and perlite in a 3:1 ratio. Six plants were planted per pot, with pot dimensions of 49 × 21 × 15 cm. The experiment included 3 independent replicates. Approximately 10 weeks after planting, once the plants had developed 5 leaves and entered the vigorous growth phase, the plants were treated with varying concentrations of MJ or SA in order to screen for effective hormone. The MJ concentrations used were 10 μM, 100 μM, 1 mM, 5 mM, and 10 mM, the solution (Aladdin, Shanghai, China) was prepared with 0.2% Tween 20 (Sangon, Shanghai, China). The SA concentrations used were 100 μM and 1 mM, and the solution (Aladdin, Shanghai, China) was prepared with 0.2% Tween 20 (Sangon, Shanghai, China). The control group was treated with a 0.2% Tween 20 aqueous solution. Hormone treatments were applied weekly and sprayed on leaves until there was runoff for a total of 8 treatments. Twenty-four hours after the final treatment, the aboveground parts of the MJ-treated plants were harvested, and measurements were taken for fresh weight, PGT density, EO content, and yield. Similarly, twenty-four hours after the final treatment, the aboveground parts of the SA-treated plants were harvested, and measurements were taken for EO content.

### 2.2 MJ treatment and transcriptomic sampling strategy

*M. arvensis* plants were cultured in a temperature-controlled tissue culture room maintained at 25 °C with 70% humidity, under a 16-hour light/8-hour dark cycle with a light density of 150 μmol·m^-2^·s^-1^. Once the plants developed the first 5 leaves, they were transitioned to continuous light conditions and pre-cultured for one week. The plants were then sprayed with 1 mM MJ solution until runoff. The MJ solution (Aladdin, Shanghai, China) was prepared with 0.2% Tween 20 (Sangon, Shanghai, China). The control group was treated with a 0.2% Tween 20 aqueous solution. The third pair of leaf samples were collected at 0 hours (CK), 4 hours (H4), 8 hours (H8), and 24 hours (H24) after MJ treatment. Three biological replicates were taken for each time point. All samples were immediately frozen in liquid nitrogen and stored at -80°C for further analysis.

### 2.3 Essential oil extraction and its component analysis

The EO of *M. arvensis* was extracted via hydro-distillation using a Clevenger apparatus. A sample of 200 g of above-ground fresh plants was subjected to hydro-distillation for 50 minutes. EO content was expressed as a percentage of fresh weight (w/w). EO yield was quantified in grams per plant. The composition of the EO was analyzed using GC-MS. An Agilent 7890B gas chromatograph coupled with a 5977A mass spectrometer (Agilent Technologies, Santa Clara, USA) was employed for the GC-MS analysis. The chromatographic separation was performed on a DB-WAX column (30 m × 0.25 mm, 0.25 µm film thickness; Agilent Technologies, Santa Clara, USA). Helium was used as the carrier gas at a flow rate of 1 mL/min. The quadrupole temperature was maintained at 150°C, and the ionization mode was electron ionization (EI+) (Mahmoud and Croteau, 2001).

### 2.4 Peltate glandular trichome density analysis

From our previous preliminary experiments, we observed that the PGT density was the highest in young leaves and the lowest in old leaves. However, despite their higher PGT density, young leaves often contain immature PGTs that have not yet synthesized or stored EO. Consequently, leaves may contain PGTs at various developmental stages and sizes, complicating accurate observation and statistical analysis. The third and fourth pairs of leaves typically have mature and rounded PGTs filled with EO. Therefore, we selected the third pair of leaves of uniform size as representative samples for PGT density analysis. Fluorescence microscopy (Olympus, Tokyo, Japan) was used to capture images of the abaxial side of each leaf, focusing on the top, middle, and bottom sections. A 10x objective lens was used for imaging, and fluorescence was induced using UV light. The quantification of PGT was performed using ImageJ software (version 1.54k, National Institutes of Health, Bethesda, USA) with appropriate image processing and analysis protocols (Yan et al., 2017). This approach ensured precise quantification of trichome density across different leaf sections.

### 2.5 Total RNA extraction, cDNA library construction, and sequencing

Total RNA from plant leaves was extracted using the Plant RNA Extraction Kit (Takara, Dalian, China) following the manufacturer’s protocol. The purity and concentration of the extracted RNA were assessed with a NanoDrop 2000 spectrophotometer (Thermo Fisher Scientific, Waltham, USA). RNA samples with an A260/A280 ratio between 1.8 and 2.0, and an A260/A230 ratio above 2.0 were selected for further analysis. The RNA sequencing was performed by Biomarker Technologies (Qingdao, China). Sequencing libraries were prepared using the NEBNext^®^ Ultra™ RNA Library Prep Kit for Illumina^®^ (New England Biolabs, Ipswich, USA) following the manufacturer’s protocol, with index codes added for sample identification. mRNA was purified from total RNA using poly-T magnetic beads. First-strand and second-strand cDNA synthesis were carried out using M-MuLV Reverse Transcriptase, DNA Polymerase I, and RNase H. After adenylation of 3’ ends, NEBNext Adaptors were ligated. cDNA fragments (~240 bp) were purified using the AMPure XP system (Beckman Coulter, Beverly, USA). The USER enzyme was applied to the adaptor-ligated cDNA before PCR amplification using Phusion High-Fidelity DNA polymerase and specific primers. Finally, PCR products were purified and the library quality was assessed on an Agilent Bioanalyzer 2100 (Agilent Technologies, Santa Clara, USA). Clean reads were obtained by processing raw fastq data with in-house scripts to remove adapters, poly-N sequences, and low-quality reads, while calculating Q20, Q30, GC content, and sequence duplication levels for quality control. Clean reads were assembled *de novo* using Trinity software (version 2.8.4) with default parameters. Functional annotation of the assembled sequences was carried out using several databases, including the NR (NCBI non-redundant protein sequences), Pfam (Protein family), KOG/COG/eggNOG (Clusters of Orthologous Groups of proteins), Swiss-Prot (A manually annotated and reviewed protein sequence database), KEGG (Kyoto Encyclopedia of Genes and Genomes), and GO (Gene Ontology), to provide comprehensive insights into the biological functions of the identified genes (Li et al., 2023).

### 2.6 Differentially expressed genes identification and pathway enrichment analysis

Differentially expressed genes (DEGs) were identified using DESeq2 software with the threshold false discovery rate (FDR) < 0.05 and |log2 Fold Change| ≥ 1 (Love et al., 2014). The GO classification analysis and KEGG pathway enrichment analysis were performed using the GO database (https://github.com/tanghaibao/Goatools) and KOBAS program (http://kobas.cbi.pku.edu.cn/) with *p* < 0.05. ClusterProfiler (version 4.12.6, Yulab, Guangzhou, China) was used to visualize the enrichment results using bar graphs (Klopfenstein et al., 2018). Volcano plots were constructed and analyzed using TBtools software (version 2.119, Guangzhou, China), allowing for the identification of significant DEGs by plotting the log2 fold change against the -log10 *p*-value (Chen et al., 2020). Veen diagram analysis was performed using the VENNY platform (https://bioinfogp.cnb.csic.es/tools/venny/index). Heatmaps were constructed from log2(TPM) values using TBtools (Chen et al., 2020).

### 2.7 Transcription factor identify, gene co-expression analysis, and promoter binding element analysis

TFs were predicted using the Biomarker Cloud platform (http://www.biocloud.net/) (Du et al., 2021). Gene co-expression analysis was conducted via the SRplot platform (http://www.bioinformatics.com.cn) (Tang et al., 2023). DEGs involved in monoterpenoid biosynthesis of *M. arvensis*, along with key enzyme genes previously reported in monoterpenoid biosynthesis pathways (Lange and Croteau, 1999b; Lange et al., 2011), and differentially expressed transcription factors (DETFs), were used to construct a co-expression trend network diagram (**Supplementary Table S1**). Eight monoterpenoid biosynthesis enzyme genes (**Supplementary Table S2**) were mapped to the transcriptome database with a reference genome by local Blast, and the promoter sequence was searched. Promoter binding elements analysis was then performed using the PlantRegMap platform (https://plantregmap.gao-lab.org/) (Du et al., 2021).

### 2.8 Weighted gene co-expression network analysis

DEGs involved in monoterpenoid biosynthesis and DETFs were used to perform weighted gene co-expression network analysis (WGCNA). The WGCNA was conducted using the Biomarker Cloud platform (http://www.biocloud.net/) (Du et al., 2021). Five co-expressed modules were identified by WGCNA. Genes in module 5 were selected to predict and analyze protein–protein interaction (PPI) using the STRING database (https://cn.string-db.org). The interactions were filtered based on a confidence score threshold of 0.7 to ensure high reliability. The resulting PPI network was then imported into Cytoscape (version 3.10.2, Cytoscape Consortium, San Diego, USA). To identify hub genes, the CytoHubba plugin was employed, and the top 15 hub genes were selected based on their MCC scores, indicating their potential key roles in monoterpenoid biosynthesis biological processes under study (Li et al., 2024).

### 2.9 Quantitative real-time PCR analysis

The total RNA of the leaf samples was extracted using the RNAprep Pure Plant Kit (Tiangen, Beijing, China). The quality and concentration of the RNA were assessed by agarose gel electrophoresis (Major Science, Saratoga, USA) and Nanodrop 2000 spectrophotometer (Thermo Fisher Scientific, Waltham, USA). The cDNA was synthesized using the HiScript II Reverse Transcriptase kit (Vazyme, Nanjing, China). The gene-specific primers were designed with Primer Premier software (version 6, PREMIER Biosoft International, Palo Alto, USA). The specific primers used are shown in **Supplementary Table S3**. Quantitative real-time PCR (qRT-PCR) was performed using AceQ qPCR SYBR Green Master Mix kit (Vazyme, Nanjing, China) and a Bio-Rad MiniOpticon Real-Time PCR machine (Bio-Rad, Hercules, USA). The PCR reaction conditions were: pre-denaturation at 95°C for 1 min, followed by denaturation at 95°C for 10 seconds, and annealing at 60°C for 30 seconds, a total of 40 cycles were performed. All the data were normalized using *Actin* gene as reference, and the gene expression level was calculated using 2^−ΔΔCT^ (Li et al., 2023).

### 2.10 Data analysis

The values are presented as the mean ± standard deviation (SD) of at least 3 replicates. One-way analysis of variance (ANOVA) was performed using GraphPad Prism (version 8.0, GraphPad Software, San Diego, USA), and statistical significance was set at *P < 0.05, **P < 0.01, ***P < 0.001, and ****P< 0.0001 (Park et al., 2024).

## 3 Results

### 3.1 Effect of methyl jasmonate on the production of essential oil and peltate glandular trichome density in *Mentha arvensis*

**Figure 1:**
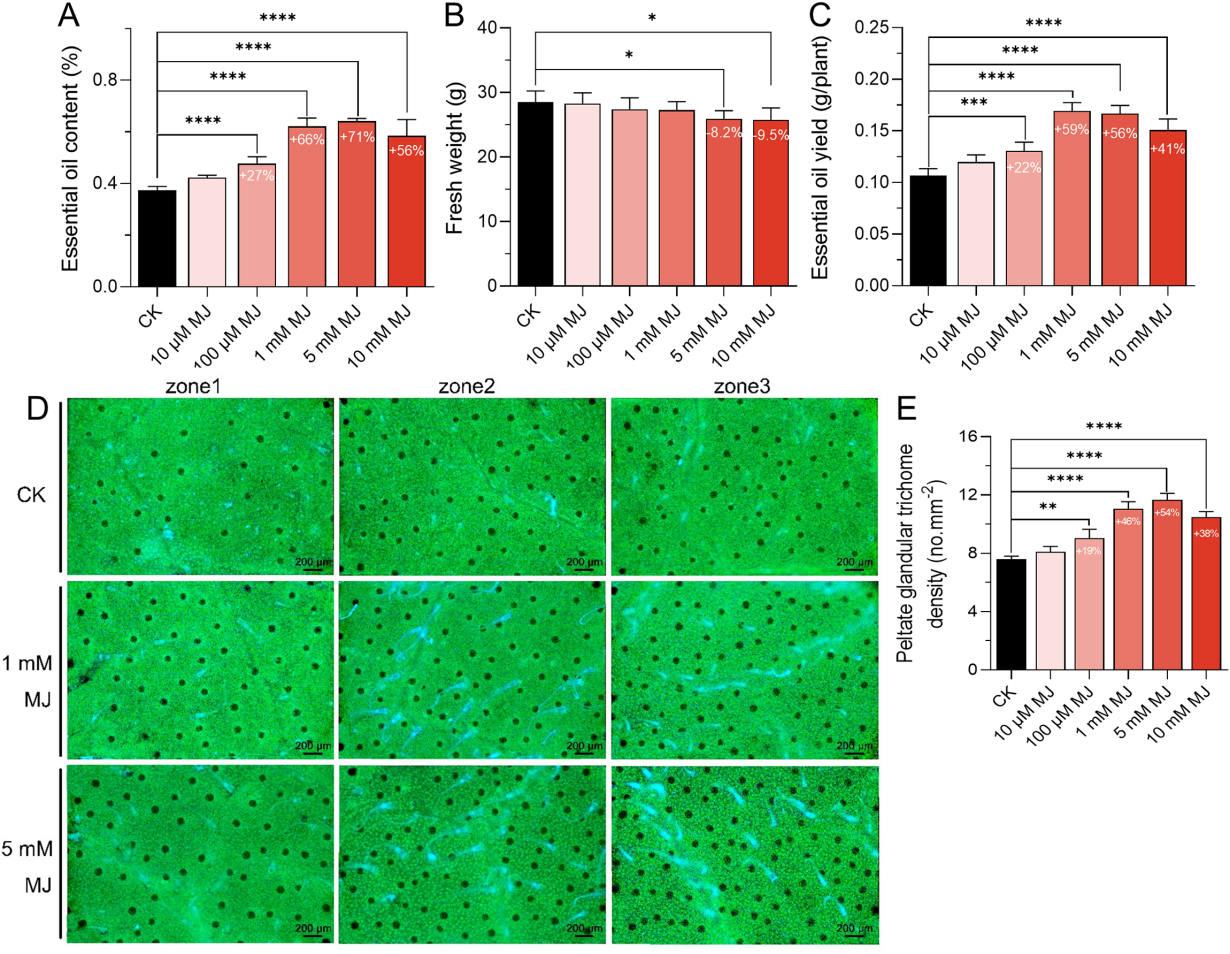
Effects of different concentrations of MJ on essential oil (EO) production and peltate glandular trichome (PGT) density. **(A)** EO content. **(B)** Plant growth. **(C)** EO yield. **(D)** PGTs were observed and recorded using a fluorescence microscope. The green background represents the autofluorescence of chlorophyll, the black dots indicate the autofluorescence of PGTs. **(E)** Histogram showing PGT density statistics. *P < 0.05, **P < 0.01, ***P < 0.001, ****P < 0.001.

The biosynthesis of metabolites in many medicinal plants is closely linked to defense mechanisms (Bednarek and Osbourn, 2009). MJ and salicylic acid (SA), two key defense-related phytohormones, were applied to mint leaves to evaluate their effects. MJ significantly increased the EO content, while SA showed minimal to no effect (**Supplementary Figure 1**). Further experiments demonstrated that MJ promoted *M. arvensis* EO synthesis in a concentration-dependent manner (**Figures 1A–C**). As the MJ concentration increased from 10 μM to 5 mM, a corresponding increase in EO content was observed. At the 5 mM dose of MJ, the EO content peaked, increasing by 71.20% compared to the control group. While high concentrations of MJ slightly reduced plant growth (e.g., a 9.51% decrease in fresh weight under 10 mM treatment), this did not hinder EO production. Notably, under 1 mM MJ treatment, the EO production increased by 58.50% compared to the control group, although the highest EO content was achieved under 5 mM MJ treatment.

PGTs are where mint EO is synthesized and stored (Croteau et al., 2005). The density of mint PGT was assessed using fluorescence microscopy. The results showed that the trends in PGT density were closely aligned with the changes in EO content (**Figures 1D, E**). Specifically, the PGT density peaked under 5 mM MJ treatment, with an increase of 53.69% compared to the control group.

### 3.2 Effects of methyl jasmonate on the essential oil composition of *Mentha arvensis*

Across all treatments, 28 chemical components were identified in *M. arvensis* EOs, representing over 98.52% of the total composition (**Table 1**). In general, the types and relative proportions of the compounds identified remained consistently similar across all treatments. In each group, monoterpenoids were the predominant components. The top 5 compounds were L-menthol (79.07%~85.86%), L-menthone (4.93%~10.89%), neomenthol (1.60%~1.90%), isomenthone (1.20%~1.52%) and pulegone (0.91%~2.66%). These compounds represented over 94.80% of the total volatiles in each treatment. However, subtle variations were observed between different groups, particularly under the 5 mM MJ treatment. L-menthol, the predominant component of *M. arvensis* EO, serves as a key quality indicator (Adlard, 2010; Wei et al., 2023). In all treatments, L-menthol content exceeded 79.07%. The control group exhibited the highest L-menthol content (85.86%). As MJ concentration increased, L-menthol content decreased slightly. Under 1 mM MJ treatment, L-menthol content was 82.57%, while at a 5 mM MJ dose, it decreased to 79.07%, a significant reduction of 7.90% compared to the control group (*p* < 0.05). In contrast, under 5 mM MJ treatment, menthone and pulegone content increased significantly by 120.80% (*p* < 0.05) and 191.72% (*p* < 0.01), respectively, compared to the control.

**Table 1:** Effects of MJ on the EO chemical composition of *Mentha arvensis*.

No.	RT	Component		Relative content (%)		
		Name	CAS	CK	1 mM MJ	5 mM MJ
1	34.34	L-menthol	002216-51-5	85.86 ± 2.27	82.57 ± 1.16	*79.07 ± 2.69 (-7.90%)
2	20.21	L-menthone	014073-97-3	4.93 ± 1.75	7.95 ± 1.00	*10.89 ± 2.84 (+120.80%)
3	31.12	Neomenthol	000491-01-0	1.90 ± 1.66	1.79 ± 0.00	1.60 ± 0.09
4	22.31	Isomenthone	000491-07-6	1.20 ± 0.24	1.42 ± 0.04	1.52 ± 0.16
5	33.13	Pulegone	000089-82-7	0.91 ± 0.21	1.69 ± 0.54	**2.66 ± 0.44 (+191.72%)
6	37.10	Germacrene D	023986-74-5	0.82 ± 0.26	/	/
7	38.09	Piperitone	000089-81-6	0.61 ± 0.04	0.54 ± 0.05	0.53 ± 0.05
8	16.43	3-Octanol	000589-98-0	0.48 ± 0.21	0.64 ± 0.02	0.65 ± 0.06
9	29.90	β-Caryophyllene	000087-44-5	0.35 ± 0.11	0.19 ± 0.09	0.12 ± 0.12
10	28.82	Isopulegol	000089-79-2	0.34 ± 0.04	0.35 ± 0.01	0.32 ± 0.03
11	36.57	Lavandulol	000498-16-8	0.26 ± 0.05	0.20 ± 0.06	0.17 ± 0.08
12	37.50	α-Terpineol	000098-55-5	0.20 ± 0.03	0.19 ± 0.01	0.17 ± 0.03
13	28.22	Isopulegone	029606-79-9	0.18 ± 0.07	0.45 ± 0.20	0.38 ± 0.07
14	38.59	Bicyclogermacrene	024703-35-3	0.15 ± 0.05	/	/
15	23.13	cis-3-Hexenyl isovalerate	035154-45-1	0.10 ± 0.01	0.11 ± 0.00	0.10 ± 0.02
16	15.64	(Z)-3-Hexen-1-ol	000928-96-1	0.04 ± 0.01	0.04 ± 0.00	0.03 ± 0.00
17	24.52	β-Bourbonene	005208-59-3	0.04 ± 0.01	/	/
18	59.29	α-Cadinol	000481-34-5	0.04 ± 0.02	0.04 ± 0.01	0.04 ± 0.01
19	8.38	Limonene	000138-86-3	0.03 ± 0.01	0.08 ± 0.02	0.10 ± 0.05
20	8.72	Eucalyptol	000470-82-6	0.03 ± 0.01	0.05 ± 0.01	0.05 ± 0.02
21	4.14	α-Pinene	000080-56-8	0.01 ± 0.01	0.03 ± 0.01	0.03 ± 0.01
22	5.90	β-Pinene	000127-91-3	0.01 ± 0.01	0.04 ± 0.01	0.04 ± 0.01
23	57.65	Spathulenol	006750-60-3	0.01 ± 0.02	0.02 ± 0.01	/
24	58.61	τ-Muurolol	019912-62-0	0.01 ± 0.01	/	/
25	59.16	cis-3-Hexenyl phenyl acetate	042436-07-7	0.01 ± 0.00	0.03 ± 0.00	0.03 ± 0.01
26	7.44	β-Myrcene	000123-35-3	0.00 ± 0.01	0.02 ± 0.00	0.01 ± 0.00
27	51.80	Caryophyllene oxide	001139-30-6	/	/	/
28	60.89	Methyl jasmonate	001211-29-6	/	/	0.02 ± 0.01
				98.52	98.74	98.79

RT, Retention time; /, Not detected or a component with a relative peak area less than 0.01%; *p < 0.05.

### 3.3 RNA sequencing data and quality assessment

Transcriptome sequencing was performed to explore how MJ regulates EO biosynthesis and PGT development in *M. arvensis* at the transcriptional level. Total RNA was extracted from *M. arvensis* leaves at 0, 4, 8, and 24 hours after MJ treatment. Twelve cDNA libraries (3 replicates per treatment) were sequenced, yielding 81.56 Gb of clean data. Each library produced no less than 6.41 Gb of clean data. GC content ranged from 46.91% to 47.38% across the 12 transcriptome samples. The percentage of Q30 bases was at least 92.58% (**Table 2**). Sample replicate correlation analysis (**Supplementary Figure S2A**) showed that the Pearson’s correlation coefficients within the same treatment group were not less than 0.899. Furthermore, the principal component analysis (**Supplementary Figure S2B**) revealed that the values of PC1 and PC2 were 17.14% and 13.23%, respectively. These results indicated that the dataset is robust, with high reproducibility between samples, making it suitable for further analysis.

**Table 2:** Statistical table of sequencing data.

Samples	Clean Reads	Clean Bases (bp)	GC (%)	Q30 (%)
CK-1	22,868,136	6,847,194,680	46.97%	92.58%
CK-2	21,848,006	6,541,720,413	46.89%	93.27%
CK-3	24,687,751	7,382,323,417	47.38%	93.98%
H4-1	22,180,033	6,639,000,553	47.20%	93.85%
H4-2	24,488,593	7,330,430,519	47.15%	93.24%
H4-3	23,378,976	7,001,160,200	47.03%	93.25%
H8-1	22,149,639	6,620,820,606	46.91%	94.00%
H8-2	22,042,766	6,594,444,350	46.77%	93.36%
H8-3	21,413,933	6,405,415,866	46.89%	93.76%
H24-1	22,954,164	6,873,251,249	46.96%	93.12%
H24-2	21,495,755	6,438,541,892	47.12%	93.13%
H24-3	22,985,286	6,882,903,469	47.23%	93.43%

CK, Control; H4, H8, H24, Sampling at 4, 8, and 24 hours after 1 mM MJ treatment; Q30, indicates the percentage of bases with a Phred value >30.

### 3.4 Functional annotation of unigenes and identification of differentially expressed genes

**Figure 2:**
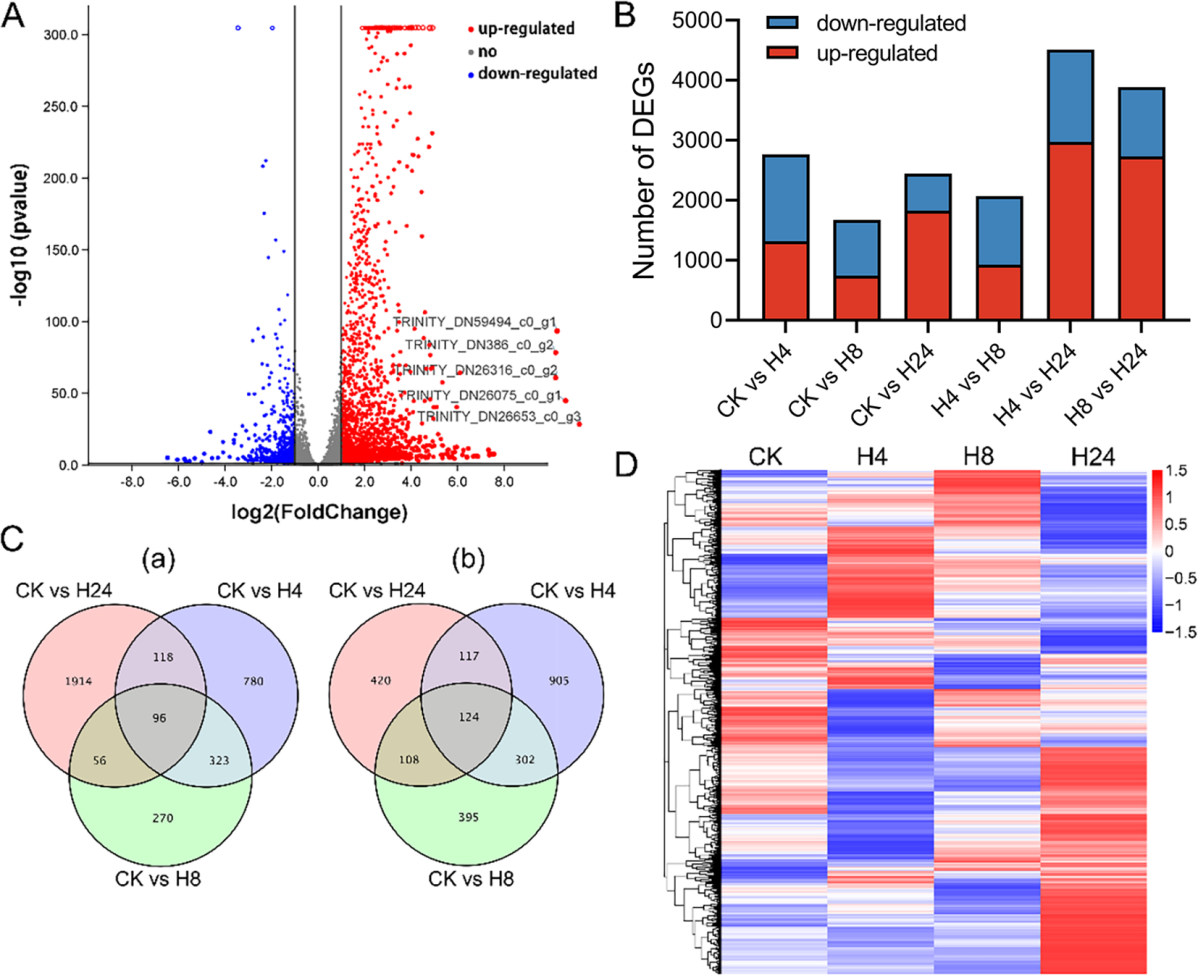
Characteristics of DEGs under MJ treatment. **(A)** Volcano plot of MJ treatment for 24 hours. **(B)** Histogram showing DEG expression changes under MJ treatment for 4, 8, and 24 hours. **(C)** Venn diagrams of up-regulated and down-regulated genes: (a) Up-regulated genes; (b) Down-regulated genes. **(D)** Heat map of DEGs.

A total of 55,154 unigenes were annotated using public protein databases (NR, Swiss-Prot, GO, COG, KOG, KEGG, etc.) with a BLAST E-value cutoff of 1.0 × 10^-5^ (**Supplementary Table S4**). To evaluate the effect of MJ on *M. arvensis* gene expression, DEG analysis was performed. A total of 7,428 DEGs were identified across six comparative groups (**Figure 2**). In the CK vs H4 comparison, 2,765 DEGs were identified, including 1,317 up-regulated and 1,448 down-regulated genes. In the CK vs H8 comparison, 1,674 DEGs were identified, including 745 up-regulated and 929 down-regulated genes. In the CK vs H24 comparison, 2,443 DEGs were identified, including 1,828 up-regulated and 615 down-regulated genes. In the H4 vs H8 comparison, 2,069 DEGs were identified, including 929 up-regulated and 1,140 down-regulated genes. In the H4 vs H24 comparison, 4,511 DEGs were identified, including 2,971 up-regulated and 1,540 down-regulated genes. In the H8 vs H24 comparison, 3,885 DEGs were identified, including 2,731 up-regulated and 1,154 down-regulated genes (**Figures 2A, B**; **Supplementary Figure S3**). In addition, a total of 4,659 DEGs were identified across the CK vs H4, CK vs H8, and CK vs H24 comparisons. The analysis revealed that 96 genes were consistently up-regulated, while 124 genes consistently down-regulated after MJ treatment (**Figure 2C**). Over time, different genes showed varied expression patterns in response to MJ (**Figure 2D**). Overall, MJ significantly affected gene transcription in *M. arvensis*.

### 3.5 GO enrichment and KEGG pathway analysis of differentially expressed genes

**Figure 3:**
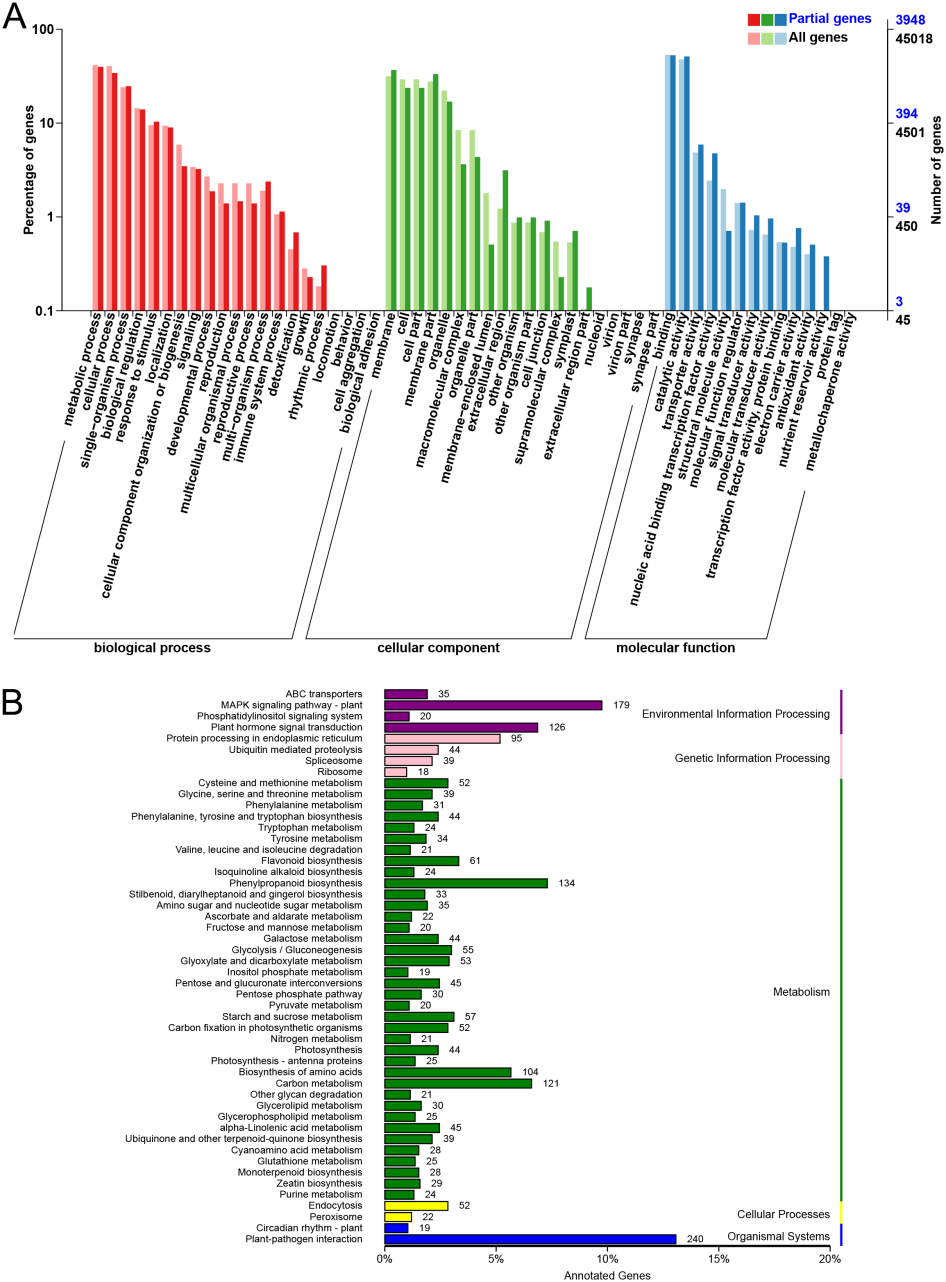
GO enrichment and KEGG pathway characterization of DEGs after MJ treatment. **(A)** GO enrichment analysis. **(B)** KEGG pathway analysis.

GO enrichment analysis was performed to explore the potential functions of these DEGs from the CK vs H4, CK vs H8, and CK vs H24 comparisons (**Figure 3**), categorizing them into 3 major groups: biological process (BP), cellular component (CC), and molecular function (MF). Within BP, the top 4 subcategories were “metabolic process”, “cellular process”, “single-organism process” and “biological regulation”. In CC, the top 3 subcategories were “membrane”, “cell” and “cell part”. In MF, most DEGs clustered in “binding”, followed by “catalytic activity” (**Figure 3A**). Notably, 1,569 DEGs were annotated in the “metabolic process” subcategory, making it the most abundant category in the GO enrichment analysis. Additionally, 409, 203, 45, 41, and 20 genes were annotated in the subcategories of “response to stimulus”, “detoxification”, “immune system process”, “signal transducer activity”, and “antioxidant activity”, respectively (**Figure 3A**; **Supplementary Table S5**). These subcategories have been shown to play key roles in plant resistance to stress.

KEGG pathway analysis showed that 130 pathways were significantly enriched under MJ treatment. DEGs were predominantly enriched in the plant-pathogen interaction (240 genes), MAPK signaling (179 genes), phenylpropanoid biosynthesis (134 genes), and plant hormone signal transduction pathways (126 genes). Notably, 28 genes were enriched in the monoterpenoid biosynthesis pathway (**Figure 3B**; **Supplementary Table S6**). Additionally, 12 genes (1.17%), 9 genes (1.53%), and 19 genes (2.13%) were enriched in this pathway in the CK vs H4, CK vs H8, and CK vs H24 comparisons, respectively (**Supplementary Figure S4**), indicating that H24 induced a higher number of genes involved in monoterpenoid biosynthesis. For example, in the CK vs H24 comparison, 3 genes encoding cytochrome P450 enzyme (CYP450) (TRINITY_DN14738_c1_g1, TRINITY_DN32509_c0_g1, and TRINITY_DN46431_c0_g1), 2 genes encoding IPR (TRINITY_DN60151_c0_g1 and TRINITY_DN90669_c0_g1), 1 gene encoding LS (TRINITY_DN5134_c0_g1), and 1 gene encoding MD (TRINITY_DN28821_c1_g1 encoded) were identified.

### 3.6 Gene expression profiles related to JA signaling and monoterpenoid biosynthesis

**Figure 4:**
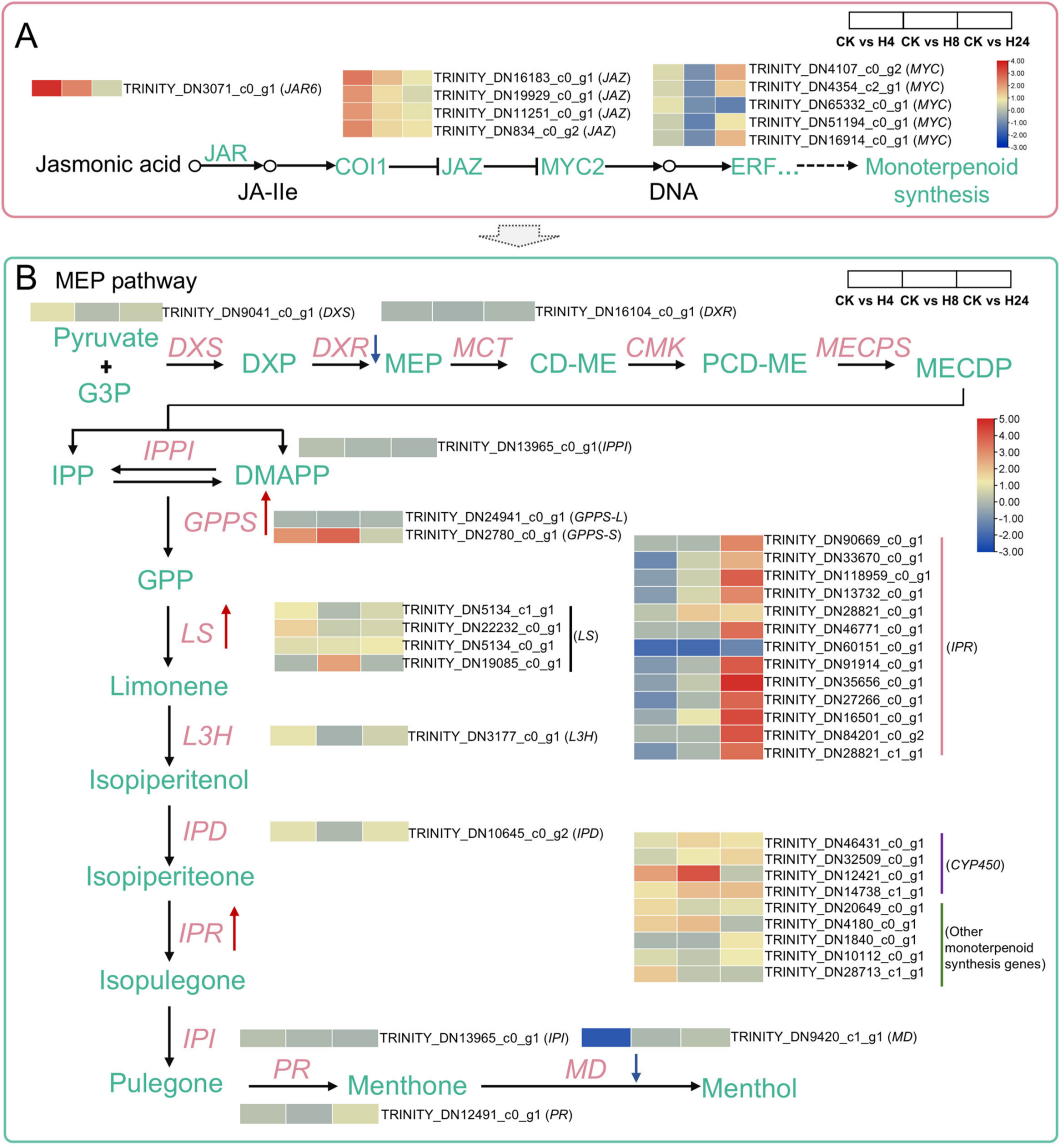
Heatmaps of the DEGs in the JA signaling and monoterpenoid biosynthesis pathways under MJ treatment. **(A)** JA signaling pathway: JAR6, Jasmonoyl-L-amino acid synthetase; COI1, Coronatine insensitive 1; JAZ, Jasmonate ZIM domain protein; MYC2, bHLH transcription factor MYC2. **(B)** Monoterpenoid biosynthesis pathway: DXS, 1-deoxy-D-xylulose-5-phosphate synthase; DXR, 1-deoxy-D-xylulose-5-phosphate reductoisomerase; MCT, 2-C-methyl-D-erythritol 4-phosphate cytidylyltransferase; CMK, 4-diphosphocytidyl-2-C-methyl-D-erythritol kinase; MECPS, 2-C-methylerythritol-2,4-cyclodiphosphate synthase; IPPI, Isopentenyl diphosphate isomerase; GPPS, Geranyl diphosphate synthase; LS, Limonene synthase; L3H, Limonene-3-hydroxylase; IPD, trans-Isopiperitenol dehydrogenase; IPR, Isopiperitenone reductase; IPI, cis-Isopulegone isomerase; PR, Pulegone reductase; MD, L-menthol dehydrogenase.

In *Arabidopsis thaliana*, the F-box protein coronatine insensitive 1 (COI1) acts as the receptor for JA. Perception of JA-Ile by the SCF^COI1^ complex triggers the degradation of JAZ proteins through the 26S proteasome. This process activates downstream TFs involved in JA responses, such as Octadecanoid responsive 3 (ORA3), a member of the APETALA2/ethylene responsive factor (AP2/ERF) family (Montiel et al., 2011; Yu et al., 2021b). In this study, 10 DEGs were identified as key factors of JA signaling in *M. arvensis* (**Figure 4A**). Among them, 1 *JAR6* gene (TRINITY_DN3071_c0_g1) and 4 *JAZ9* genes (TRINITY_DN16183_c0_g1, TRINITY_DN19929_c0_g1, TRINITY_DN11251_c0_g1 and TRINITY_DN834_c0_g2) were significantly up-regulated at 4 hours after MJ treatment. Two *MYC2* genes (TRINITY_DN4107_c0_g2 and TRINITY_DN16914_c0_g1) were significantly up-regulated at 24 hours after MJ treatment (**Figure 4A**; **Supplementary Table S1**).

In *M. arvensis*, monoterpenoids are the primary components of its EOs. KEGG enrichment results indicated that “Monoterpenoid biosynthesis” was one of the most significantly enriched pathways under MJ treatment. Twenty-eight DEGs associated with monoterpenoid biosynthesis were identified in response to MJ treatment. Notably, *GPPSs* were significantly up-regulated at 4 and 8 hours after MJ treatment, 4 *LS* genes were up-regulated both at 4, 8 and 24 hours, and 12 *IPR* genes were significantly up-regulated at 24 hours after MJ treatment. CYP450, catalyze the decoration of terpenoid basic skeletons and thereby contribute significantly to their structural diversity (Weitzel and Simonsen, 2015). In this study, 4 *CYP450* genes were identified as significantly expressed (**Figure 4B**; **Supplementary Table S1**). L-menthol is the predominant component of *M. arvensis* EO, which is synthesized through a series of enzymatic reactions. The enzymatic catalysis mechanism of L-menthol synthesis has been extensively studied in 3 *Mentha* varieties: *Mentha piperita*, *Mentha spicata*, and *Mentha haplocalyx* (Ahkami et al., 2015). Using reference genes from these varieties as queries, 11 orthologous genes were identified in *M. arvensis* (**Supplementary Table S1**), including the previously reported *LS* and *MD* genes (Akhtar et al., 2017; Wang et al., 2013).

### 3.7 Identification of DETFs and co-expression analysis with monoterpenoid biosynthesis genes

**Figure 5:**
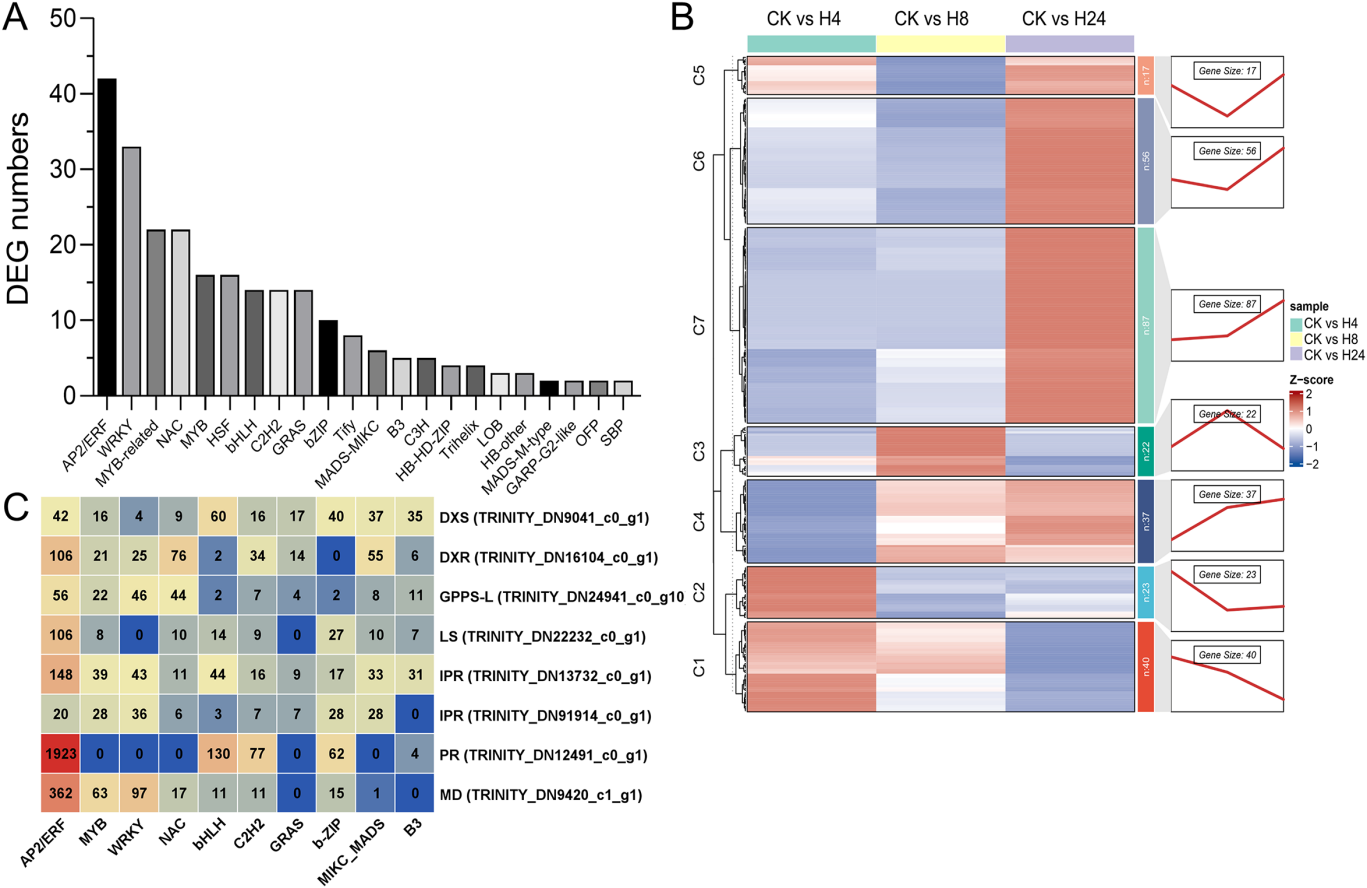
Analysis of the co-expression patterns of monoterpenoid biosynthesis genes and differentially expressed transcription factors (DETFs). **(A)** Identification of DETFs. **(B)** Gene clusters classified based on co-expression patterns. **(C)** Statistics of promoter binding elements for key enzyme genes.

TFs regulate plant growth, development, and secondary metabolite synthesis (Yang et al., 2012). In this study, 260 DETFs belonging to 22 families were identified after MJ treatment for 4, 8, and 24 hours (**Figure 5A**; **Supplementary Table S1**). The top 8 families with the highest number of DEGs were AP2/ERF (42 genes), MYB (including MYB-related, 38 genes), WRKY (33 genes), NAC (22 genes), HSF (heat shock transcription factors, 16 genes), bHLH (14 genes), C2H2 (C2H2 zinc-finger protein, 14 genes), and GRAS (GAI-RGA-and-SCR, 14 genes). These TFs responded positively to MJ treatment. To further explore the regulatory relationship between TFs and monoterpenoid biosynthesis, a co-expression pattern analysis of DETFs and monoterpenoid biosynthetic genes was performed (**Figure 5B**). The analysis identified 7 gene clusters. Each cluster exhibited a unique expression pattern, indicating a close association between TFs and monoterpenoid biosynthetic enzyme genes. Most of the highly expressed monoterpenoid biosynthesis genes (Log2FC > 3) were grouped into cluster 6. Therefore, further analysis focused on this cluster. In cluster 6, the top 3 TF families with the highest number of DEGs were AP2/ERF (17 genes), WRKY (16 genes), and MYB (10 genes) (**Figure 5B**; **Supplementary Table S2**). In this study, 8 monoterpenoid biosynthesis genes, including *DXS*, *DXR*, *GPPS-L*, *LS*, *IPR, PR*, and *MD*, which are either highly expressed genes (Log2FC > 3) or key genes in this process as previously reported (Lange and Croteau, 1999a; Lange et al., 2011; Mahmoud et al., 2004) were selected for promoter analysis. These genes contained the highest number of AP2/ERF binding sites (2,763), followed by bHLH (266), WRKY (251), and MYB (197) (**Figure 5C**; **Supplementary Table S2**).

### 3.8 WGCNA of differentially expressed monoterpenoid biosynthesis genes and transcription factors

**Figure 6:**
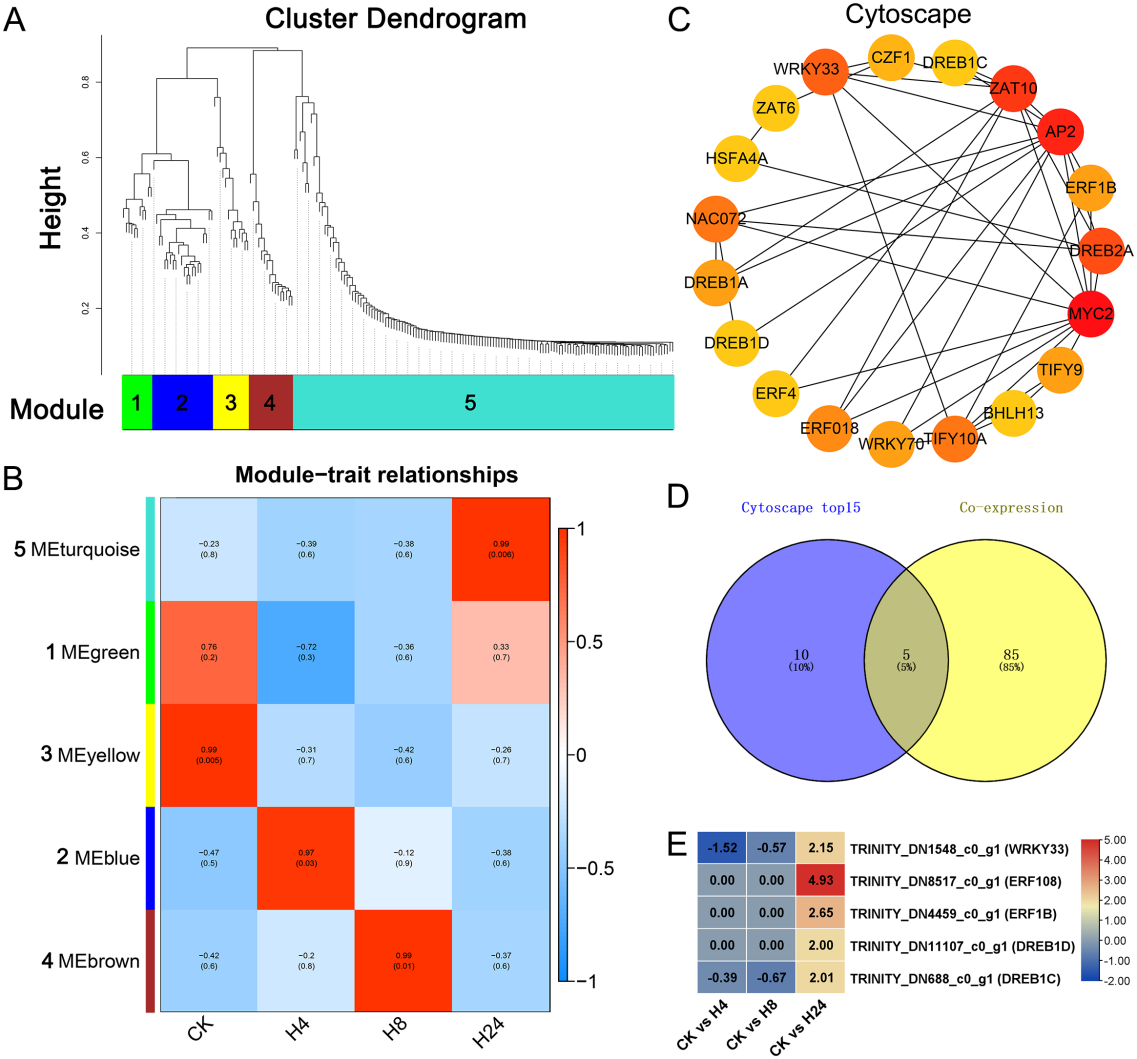
WGCNA of differentially expressed monoterpenoid biosynthesis genes and transcription factors. **(A)** Hierarchical clustering of differentially expressed gene modules. **(B)** Module-trait relationships. **(C)** Cytoscape visualization of the top 15 connectivity pairs in module 5. **(D)** Venn diagram analysis of common genes between module 5 from WGCNA and cluster 6 from co-expression analysis. **(E)** Gene expression analysis of common genes identified in the Venn diagram analysis.

WGCNA was employed to construct a gene co-expression network, aiming to identify DETFs involved in MJ-induced monoterpenoid biosynthesis. Hierarchical clustering identified 5 co-expressed modules (**Figure 6A**). Most of the high expressed monoterpenoid biosynthesis genes (10 out of 11, Log2FC > 3) peaked at 24 hours after MJ treatment and were classified in module 5 (**Figure 6B**; **Supplementary Table S7**). KEGG analysis was performed for this module. Among these, 18 unigenes were annotated in the plant hormone signal transduction pathway (35.29%), 16 unigenes in the plant pathogen interaction pathway (31.37%), and 11 unigenes in the monoterpenoid biosynthesis pathway (21.57%) (**Supplementary Figure S4**). Additionally, module 5 was highly associated with the H24 group (R = 0.99, *p* = 0.006). These results closely mirrored the analysis of the monoterpenoid biosynthesis pathway in **Figure 4**, prompting a focus on module 5 (**Figure 6B**; **Supplementary Table S7**). Furthermore, all genes of module 5 were used to conduct PPI analysis, the results were visualized using Cytoscape, and hub genes were identified using CytoHubba (**Figure 6C**). A venn diagram analysis was used to identify common genes between the top 15 hub genes from the CytoHabba analysis and cluster 6 genes from the co-expression analysis (Log2FC > 1, FPKM > 10) (**Figure 5B**), which were considered as candidate genes. The results revealed 5 common genes, including 4 AP2/EFR genes—TRINITY_DN8517_c0_g1 (*ERF108*), TRINITY_DN4459_c0_g1 (*ERF1B*), TRINITY_DN11107_c0_g1 (*DREB1D*), TRINITY_DN688_c0_g1 (*DREB1C*), and 1 WRKY gene TRINITY_DN1548_c0_g1 (*WRKY33*), which were significantly up-regulated (Log2FC > 2) at 24 hours after MJ treatment (**Figures 6D, E**).

### 3.9 Validation of RNA-seq data

**Figure 7:**
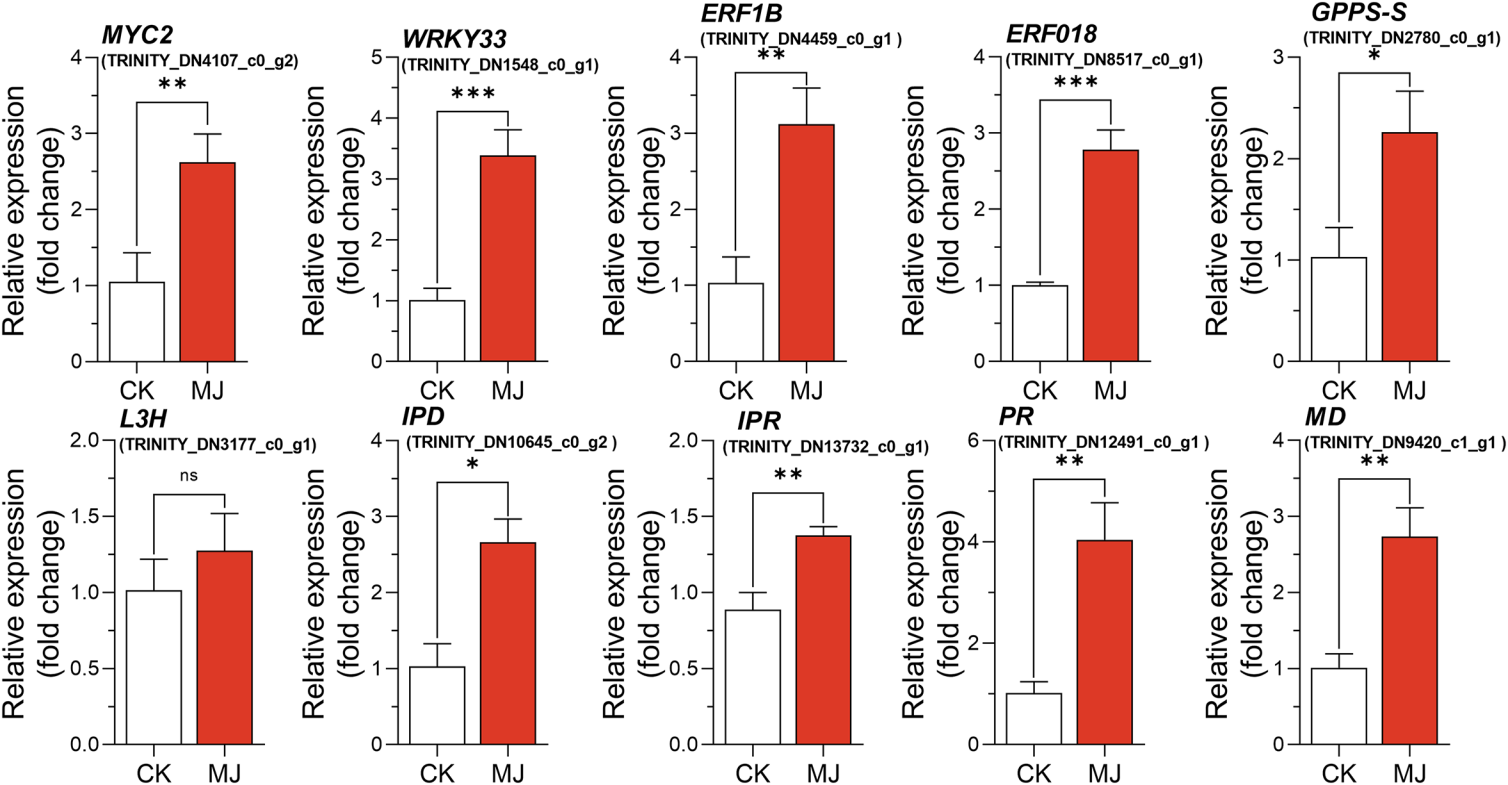
Quantitative real-time PCR verification of the RNA-seq data of 10 selected genes in the MJ treatment group (H24) and the control group (CK). *P < 0.05, **P < 0.01, ***P < 0.001.

A qRT-PCR assay with independent samples from the control and MJ treatment group (H24) was conducted to verify the expression changes of several key genes involved in monoterpenoid biosynthesis. In total, 10 genes, including 6 from the monoterpenoid biosynthesis pathway, and 4 TFs were selected to confirm the RNA-seq data. The expression levels of these selected genes, as determined by qRT-PCR, were generally consistent with the RNA-seq results (**Figure 7**).

## 4 Discussion

The EO of *M. arvensis*, rich in monoterpenoids like L-menthol and menthone, is highly valued for its medicinal properties and is widely used in industries such as medicine, cosmetics, and food (Kumar et al., 2012; Souza et al., 2020). Previous studies have shown that exogenous defense hormones such as MJ and SA can promote the biosynthesis of plant secondary metabolites including terpenoids, anthocyanins and alkaloids (Kumari et al., 2018; Li et al., 2024; Xiang et al., 2015). In some cases, these hormones also increase the density of PGT, as observed in *Artemisia annua* (Kumari et al., 2018). In our study, MJ treatment significantly enhanced EO content in *M. arvensis*, whereas SA showed minimal to no effect (**Supplementary Figure S1**). Although SA is known to antagonize JA signaling pathways and can reduce JA levels, such as in *Arabidopsis thaliana* (Barbara and David, 2002), its lack of impact on EO content in *M. arvensis* may explained by several factors. First, plant genotype plays a critical role. For example, in *Pyrus pyrifolia* (pear), exogenous SA treatment had minimal effect on the total amount of cuticle wax in leaves (mainly composed of terpenoids), suggesting insensitivity of terpenoid biosynthesis to SA in this species (Wu et al., 2018). Second, EO synthesis in *M. arvensis* may involve mechanisms beyond trichome development, which could make it less responsive to SA-induced interference (Han et al., 2022). Third, the dose, frequency, and application method of SA are important factors. Studies have shown that applying SA once or twice can promote the accumulation of terpenoid lactones, while increasing the frequency to three or four applications suppresses their synthesis (Wang et al., 2021a). Lastly, it remains necessary to confirm the changes in endogenous levels of these hormones following JA and SA treatments in this study to fully understand their interactions.

JA plays a critical role in increasing secondary metabolite production in aromatic and medicinal plants (Patt et al., 2018; Wasternack and Strnad, 2019). For example, in *Picea abies*, JA treatment induced the accumulation of monoterpenoid and diterpene in resin ducts (Schmidt et al., 2011). Similarly, MJ treatment increased the artemisinin content by about 300% in *A. annua* (Kumari et al., 2018; Maes et al., 2011) and the EO content by 36.80% in *Ocimum basilicum* (Zlotek et al., 2016). Furthermore, MJ promotes trichome development, not only in both non-glandular trichome-bearing species like *Arabidopsis thaliana* and *Medicago truncatula* (Maes and Goossens, 2010; Traw and Bergelson, 2003), but also in glandular trichome-producing species such as *Solanum lycopersicum* and *A. annua* (Maes and Goossens, 2010; Maes et al., 2011). In this study, PGT density was increased by 53.69% under 5 mM MJ treatment in *M. arvensis*. These results suggest that JA is a potent inducer of EO production through stimulating both monoterpenoid biosynthesis and PGT development in *M. arvensis*. Although MJ significantly increased the EO content, it also inhibited mint growth in a concentration-dependent manner. Treatment with 10 mM MJ reduced the fresh weight of *M. arvensis* by 9.51% (**Figure 1B**), consistent with previous studies indicating that excessive JA accumulation can suppress gibberellin biosynthesis, thereby delaying plant growth (Heinrich et al., 2013). In this study, while high concentrations of MJ slightly reduced L-menthol content, the 1 mM MJ treatment achieved the highest EO yield without significantly affecting L-menthol levels. These findings highlight the importance of optimizing MJ doses to maximize EO production (**Table 1**).

To further elucidate the molecular mechanisms underlying MJ’s effects on EO biosynthesis, RNA-seq analysis was conducted. A total of 7,428 DEGs were identified across six comparison groups (**Figure 2**). Interestingly, the observed increase in DEGs between H4 vs H24 compared to the CK vs H24, suggests a dynamic transcriptional response to MJ (**Figure 2B**). This pattern aligns with the known rapid and transient nature of JA-induced gene expression. Previous studies have shown that JA signaling triggers immediate transcriptional changes, peaking within a few hours of treatment, followed by a decline as the response stabilizes (Hickman et al., 2017). In our study, the H4 sample likely captures this peak transcriptional activity, encompassing genes involved in defense responses, secondary metabolite biosynthesis, and stress signaling pathways. By 24 hours (H24), many of these responses may have been downregulated due to feedback mechanisms or metabolic adjustments, resulting in fewer DEGs when compared to CK. For example, some core factors of JA signaling were highly expressed in H4, while some were decreased in H24 (**Figure 4**). GO enrichment analysis revealed that MJ treatment significantly influenced metabolic processes, with DEGs predominantly involved in the “response to stimulus”, “detoxification”, “immune system process”, and “signal transducer activity” categories (**Figure 3A**; **Supplementary Table S2**), which are crucial for plant responses to biotic and abiotic stress (Chi et al., 2018; Li et al., 2013; Nie et al., 2020). KEGG analysis revealed that DEGs were enriched in key pathways such as plant-pathogen interaction, MAPK signaling, phenylpropanoid biosynthesis, plant hormone signal transduction, and monoterpenoid biosynthesis (**Figure 3B**; **Supplementary Table S6**). These results indicate that JA may enhance terpenoid production in plants to effectively respond to environmental challenges (Unsicker et al., 2009).

The core elements of JA signaling were significantly differentially expressed in this study, including 1 *JAR6*, 4 *JAZ9*, and 2 *MYC2* genes (**Figure 4A**; **Supplementary Table S1**). The result is consistent with previous research in *Chrysanthemum indicum* var. *aromaticum* (Gao et al., 2020), indicating that MJ mediates JA signaling pathways, thereby regulating a range of downstream genes in *M. arvensis*. Monoterpenoids, the primary constituents of *M. arvensis* EO, are crucial for plant defense against pathogens and herbivores (Unsicker et al., 2009). KEGG analysis indicated that MJ treatment triggered significant differential expression of 28 genes involved in monoterpenoid biosynthesis and 12 genes related to terpenoid backbone biosynthesis, including genes encoding GPPS, LS, IPR, MD, and CYP450 enzymes (**Figure 4B**; **Supplementary Tables S1**, **S6**). Notably, early-stage genes such as *DXS* showed peak expression at H4, intermediate-stage genes like *GPPS* and *LS* peaked at H8, and late-stage genes like *IPR* and *MD* reached their highest expression at H24 (**Figure 4B**; **Supplementary Figure S1**). These findings align with previous studies on peppermint—in the later stages of monoterpenoid biosynthesis, L-menthol becomes the predominant component. Correspondingly, structural genes involved in the later reactions, such as *MD*, exhibit a delayed developmental timeline compared to the earlier enzymes in the monoterpenoid biosynthesis pathway (Turner et al., 2000).

The production and the accumulation of terpenoids, meticulously controlled in plants in a spatiotemporal manner, are orchestrated by TFs (Singh et al., 2024). In this study, 260 DETFs belonged to 22 families, including AP2/ERF, WRKY, MYB, and bHLH etc., were identified in *M. arvensis* after MJ treatment (**Figure 5A**, **Supplementary Table S1**). Many TFs have been reported to regulate the terpenoid biosynthesis, including bHLH, MYB, WRKY, AP2/ERF, TCP, bZIP, and NAC in previous studies (Su et al., 2019; Zhang et al., 2022). These TFs enhance terpenoid accumulation by directly or indirectly promoting the transcriptional activation of both TF and structural genes. For instance, AtMYB21 in *Arabidopsis thaliana* and FhMYB21 in *Freesia hybrida* promote terpenoid accumulation by binding to *TPS* promoters (Yang et al., 2020). In *A. annua*, bHLH1 and bHLH112 significantly promote the accumulation of sesquiterpene artemisinin by binding to the promoters of the artemisinin synthesis genes and the transcription factor *ERF1*, respectively (Ji et al., 2014; Xiang et al., 2019). In *Phalaenopsis orchids*, bHLH4 increases the monoterpenoid content approximately 950-fold by activating the *GPPS* promoter (Chuang et al., 2018). In *Dendrobium officinale*, bHLH4 and in *Arabidopsis thaliana*, MYC2 promote terpenoid synthesis by binding to the *TPS* gene (Hong et al., 2012; Yu et al., 2021c). In *Catharanthus roseus* and *A. annua*, MYC enhances terpenoid synthesis by regulating AP2/ERF and WRKY transcription factors (Chen et al., 2017; Paul et al., 2017). In *A. annua*, under JA induction, *WRKY9* activates the transcription of *GSW1*, thereby positively regulating artemisinin synthesis (Fu et al., 2021; Jiang et al., 2016). AP2/ERF transcription factors *AaERF1*, *AaERF2*, *AaTAR1* and *AaORA* are induced by JA and positively regulate artemisinin synthesis by binding to the promoter of artemisinin synthesis genes (Tan et al., 2015; Yu et al., 2012). NACs in *A. annua* and *Actinidia arguta* can both promote monoterpenoid synthesis by activating *TPS* expression (Nieuwenhuizen et al., 2015). Despite significant progress in understanding the molecular mechanisms of plant terpenoid biosynthesis, certain key mechanisms remain poorly understood. Additionally, different TFs have specific roles in regulating terpenoid biosynthesis in various plants, and these regulatory mechanisms may vary between species (Singh et al., 2024). In this study, co-expression analysis revealed that AP2/ERF, WRKY, and MYB families were strongly associated with monoterpenoid biosynthesis genes (**Figure 5B**; **Supplementary Table S2**). Promoter analysis indicated that key monoterpenoid biosynthesis genes contained binding sites for these TFs (**Figure 5C**), suggesting that AP2/ERF, WRKY, and MYB may play important roles in regulating MJ-induced monoterpenoid biosynthesis in *M. arvensis*. Furthermore, WGCNA, PPI, and CytoHubba analysis revealed that MYC2 is positioned at the center of the regulatory network and is involved the biosynthesis of various secondary metabolites, such as anthocyanins and terpenoids (Luo et al., 2023). A venn analysis identified 5 common genes between cluster 6 of the co-expression analysis and module 5 of WGCNA, including 4 AP2/ERF genes and 1 WRKY gene (**Figure 6**). Among them, ERF1B is homologous to *CrORCA3* from *Catharanthus roseus* (Montiel et al., 2011). The WRKY33 homolog from *Arabidopsis thaliana* has been reported to participate in defense responses and positively regulate the synthesis of the secondary metabolite camalexin (Zhou et al., 2020). These findings suggest that AP2/ERF TFs play an important role in regulating mint monoterpenoid biosynthesis. However, the functions of these candidate genes require further verification.

Trichomes are specialized structures formed by the highly differentiated epidermal cells of plant’s aboveground organs. Depending on their secretory function, trichomes can be divided into glandular and non-glandular types (Dai et al., 2010). The mint EO is synthesized and stored exclusively in the peltate glandular trichome (Croteau et al., 2005). Trichome development is regulated by complex molecular networks (Han et al., 2022). Currently, the molecular regulatory mechanisms governing non-glandular trichome development in plants are relatively well-understood (Han et al., 2022; Wang et al., 2021b). In plants like *Arabidopsis thaliana*, key regulatory pathways for non-glandular trichome development have been identified, involving TFs such as GLABRA1 (GL1), GLABRA3 (GL3), and TRANSPARENT TESTA GLABRA1 (TTG1). These TFs form the MYB-bHLH-WD40 (MBW) complex (Pesch and Hulskamp, 2009; Zhao et al., 2008), which activates downstream genes *GLABRA2* (*GL2*) and *TRANSPARENT TESTA GLABRA2* (*TTG2*), thereby initiating trichome differentiation (Han et al., 2022; Wang and Chen, 2008). In contrast, negative regulators such as R3-MYB transcription factors TRICHOMELESS (TCL), TRIPTYCHON (TRY), CAPRICE (CPC), and ENHANCER OF TRY AND CPC (ETC), competitively bind to TTG1-GL3/EGL3 complex, forming an inactive complex that inhibits the expression of downstream genes *GL2* and *TTG2* (Esch et al., 2003; Zhao et al., 2008). However, research on the molecular regulatory networks involved in glandular trichome development remains limited (Chalvin et al., 2020). Some progress has been made in species like *A. annua* and *Solanum lycopersicum*, where key regulatory pathways have been identified (Chalvin et al., 2020). In *A. annua*, AaMIXTA1 (MYB) interacts with AaHD8 (HD-ZIP) forming a complex that activates the downstream *AaHD1* (HD-ZIP) and *AaGSW2* (WRKY), then promoting glandular trichome initiation (Xie et al., 2021b; Yan et al., 2017, 2018). However, attempts to overexpress *MIXTA* from *Antirrhinum majus* in *Arabidopsis thaliana* failed to rescue the trichome loss phenotype, and overexpression of non-glandular trichome regulators gene *AtGL1* from *Arabidopsis thaliana* in *Solanaceae* species did not induce glandular trichome initiation, suggesting distinct regulatory pathways for glandular and non-glandular trichomes (Payne et al., 1999).

However, little is known about the molecular mechanisms underlying PGT development in the *Mentha* genus (Tissier, 2012). Recent studies have reported that in *Mentha canadensis*, the HD-ZIP transcription factor McHD-ZIP3 interacts with McMIXTA to form a complex that promotes PGT development (Qi et al., 2022). In this study, we identified homologous genes involved in non-glandular trichome development from *Arabidopsis thaliana* in *M. arvensis* transcriptome, including positive regulators like *GL3*, *GL2*, and *TTG2*, as well as negative regulators like *TRY, CPC*, and *ETC3*. These genes were significantly differentially expressed after MJ treatment (**Supplementary Figure 6**, **Supplementary Table S9**). Unfortunately, through local BLAST, we did not find homologous genes for the positive regulators *AaMIXTA*, *AaHD8*, and *AaHD1* from *A. annua*. However, homologs of *McMIXTA* and *HD-ZIP3* from *Mentha canadensis* were identified but did not show significant differential expression compared to the control, possibly due to the differences in the leaf age of the samples. Studies have reported that genes related to PGT initiation in mint, like *McMIXTA*, are highly expressed in young leaves, while the leaves collected in this experiment were mature (Qi et al., 2022). Further investigation is needed to validate the role of these candidate genes in PGT formation in mint.

## 5 Conclusion

Exogenous application of MJ enhanced the EO accumulation and increased the PGT density in *M. arvensis*. RNA-seq analysis indicated that numerous unigenes were differentially expressed, particularly those involved in JA signal transduction, monoterpenoid biosynthesis, and TFs. MJ treatment up-regulated genes associated with JA signal transduction, including *JAZs* and *MYCs*, as well as key structural genes in the monoterpenoid biosynthesis pathway, such as *GPPSs*, *LSs*, *L3Hs*, and *IPRs*. Additionally, TFs such as AP2/ERF, WRKY, MYB, and bHLH were identified as potential regulators of monoterpenoid biosynthesis or PGT development. Several key candidate genes which regulate monoterpenoid biosynthesis and PGT development were identified, even though their specific functions require further validation. These findings provide valuable insights into the molecular mechanisms governing monoterpenoid biosynthesis and PGT development, and offer potential strategies for enhancing EO production in *M. arvensis*.

## Data Availability

The data presented in the study are deposited in the NCBI repository, accession number PRJNA1202940, for more information regarding our data policies, refer to our guidelines.
