# Supplementary material for: Transcriptome analysis reveals regulatory mechanism of methyl jasmonate-induced monoterpenoid biosynthesis in Mentha arvensis L
Source: Front Plant Sci. 2025 Jan 15;15:1517851. doi: 10.3389/fpls.2024.1517851 (PMC11782960; doi:10.3389/fpls.2024.1517851)
Supplement: Supplementary file 1 [file DataSheet1.pdf]

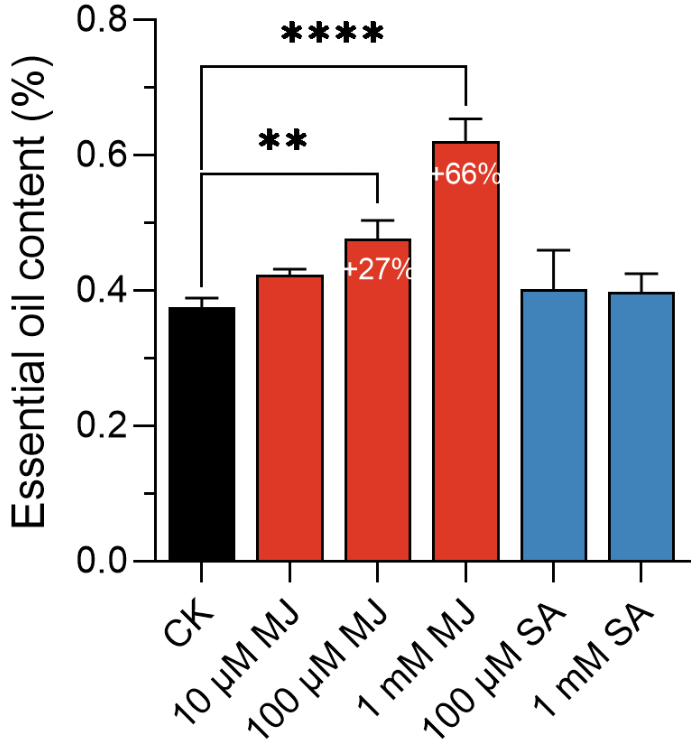

Supplementary Figure S1. Effects of methyl jasmonate (MJ) and salicylic acid (SA) on the essential oil content of *Mentha arvensis* L. *P < 0.05.
