# Supplementary material for: Transcriptome analysis reveals regulatory mechanism of methyl jasmonate-induced monoterpenoid biosynthesis in Mentha arvensis L
Source: Front Plant Sci. 2025 Jan 15;15:1517851. doi: 10.3389/fpls.2024.1517851 (PMC11782960; doi:10.3389/fpls.2024.1517851)
Supplement: Supplementary file 2 [file DataSheet2.pdf]

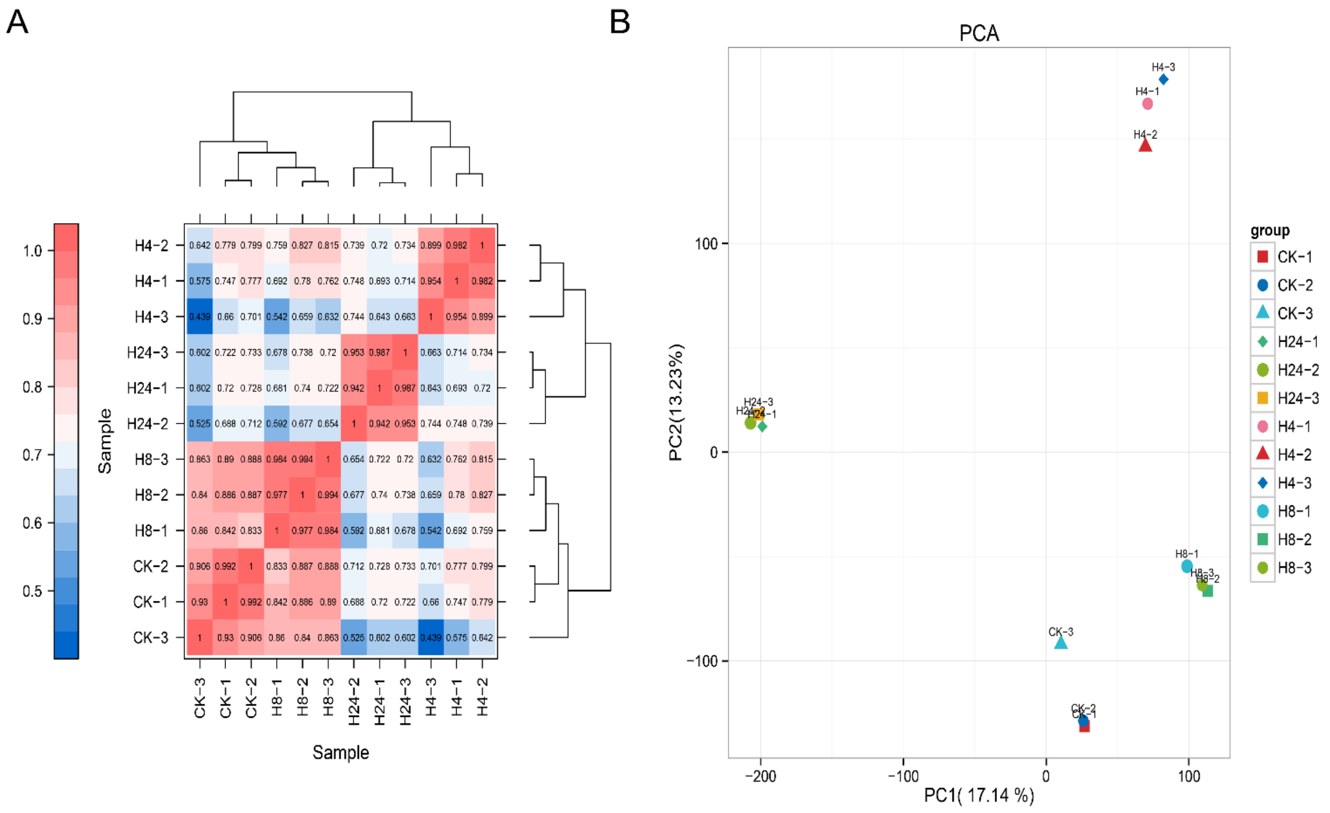

Supplementary Figure S2. Repeatability analysis between samples. (A) Heat map of expression correlation between samples. (B) Principal component analysis (PCA).
