# Supplementary figures and images for: Transcriptome analysis reveals regulatory mechanism of methyl jasmonate-induced monoterpenoid biosynthesis in Mentha arvensis L
Source: Front Plant Sci. 2025 Jan 15;15:1517851. doi: 10.3389/fpls.2024.1517851 (PMC11782960; doi:10.3389/fpls.2024.1517851)

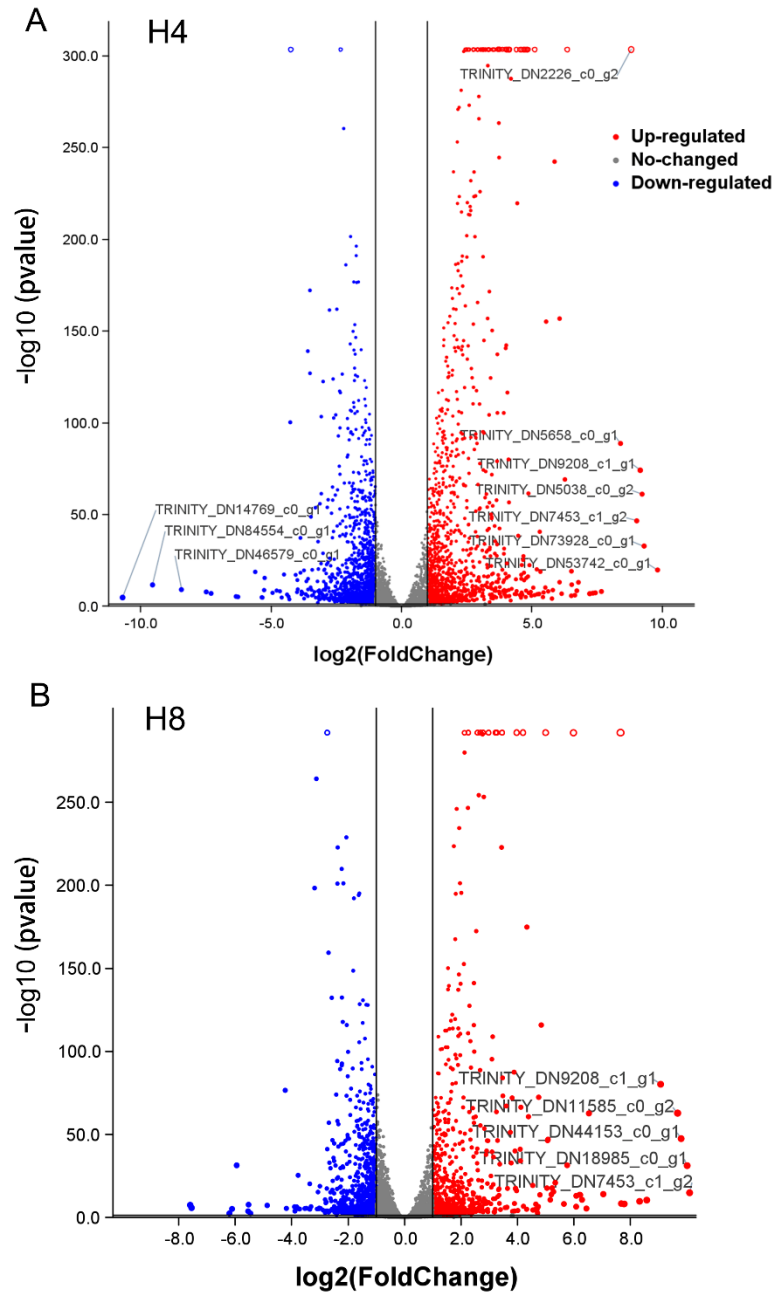

**Supplementary Figure S3.** Volcano plot showing the DEGs after MJ treatment for 4 (A) and 8 hours (B).

Supplement: Supplementary file 3 [file DataSheet3.pdf]
