# Supplementary material for: Transcriptome analysis reveals regulatory mechanism of methyl jasmonate-induced monoterpenoid biosynthesis in Mentha arvensis L
Source: Front Plant Sci. 2025 Jan 15;15:1517851. doi: 10.3389/fpls.2024.1517851 (PMC11782960; doi:10.3389/fpls.2024.1517851)
Supplement: Supplementary file 6 [file DataSheet6.pdf]

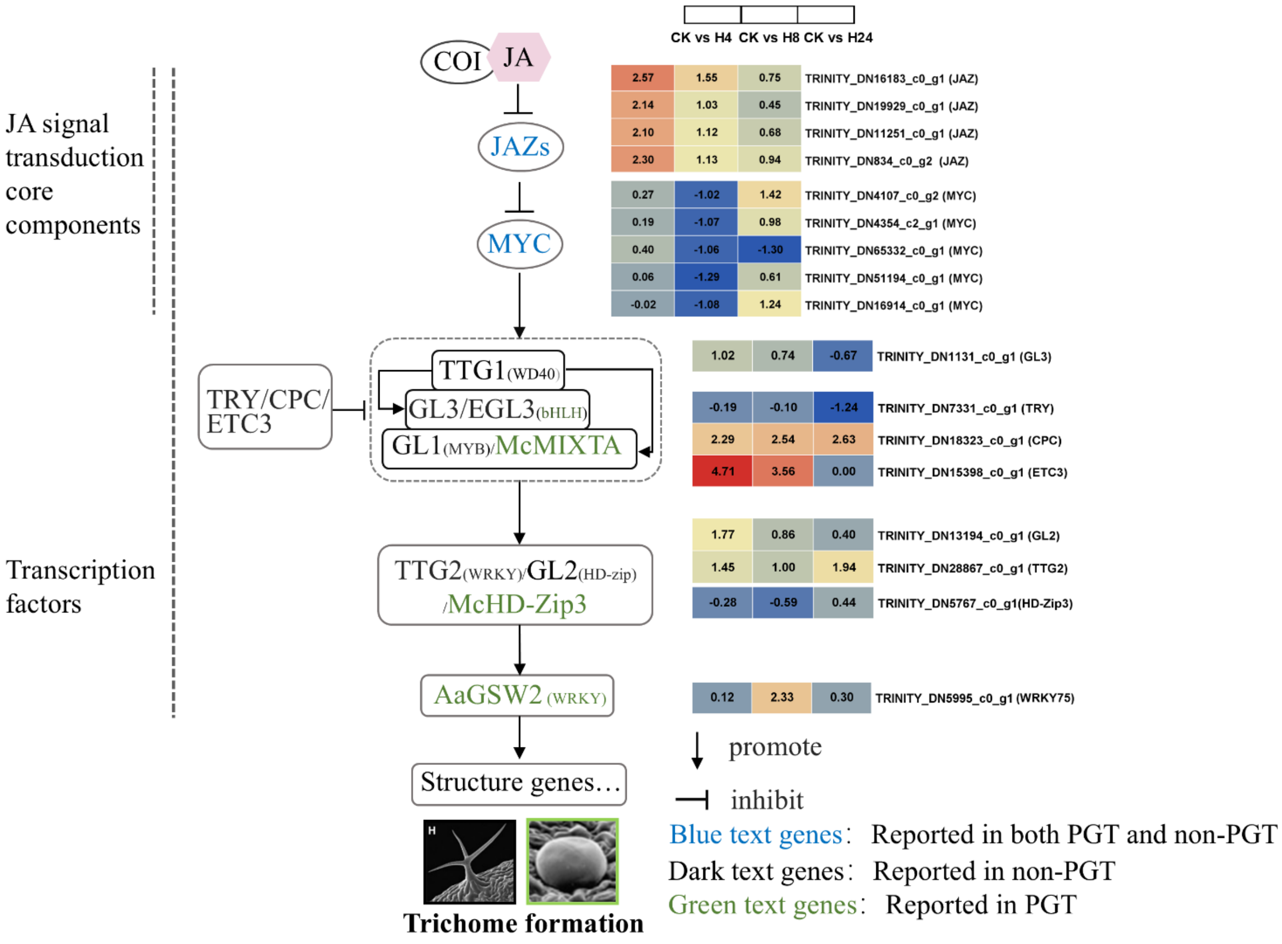

Supplementary Figure S6. Hypothesis of the model for JA signaling to regulate glandular trichome development, and the heatmaps of the DEGs in the JA signaling and trichome development under MJ treatment.
